# Surgical Complexity and Perioperative Feasibility Following Neoadjuvant Chemotherapy, Chemoradiotherapy, or Chemoimmunotherapy in Stage II–III Non-Small Cell Lung Cancer: A Retrospective Single-Institution Study

**DOI:** 10.3390/cancers18162716

**Published:** 2026-08-21

**Authors:** Hiroki Sakai, Takayuki Hatakeyama, Takahiro Homma, Kanji Otsubo, Norifumi Kakizaki, Hideki Marushima, Koji Kojima, Kei Morikawa, Naoki Furuya, Masamichi Mineshita, Yoshiya Sugiura, Junki Koike, Hisashi Saji

**Affiliations:** 1 Departments of Thoracic Surgery, St. Marianna University School of Medicine, Kawasaki 216-8511, Kanagawa, Japan; h2sakai@marianna-u.ac.jp (H.S.); takayuki.hatakeyama@marianna-u.ac.jp (T.H.); takahiro.homma@marianna-u.ac.jp (T.H.); kanji.otsubo@marianna-u.ac.jp (K.O.); norifumi.kakizaki@marianna-u.ac.jp (N.K.); kitamarushi@gmail.com (H.M.); k2kojima@marianna-u.ac.jp (K.K.); 2 Division of Respiratory Medicine, Department of Internal Medicine, St. Marianna University School of Medicine, Kawasaki 216-8511, Kanagawa, Japan; mokke@marianna-u.ac.jp (K.M.); n2furuya@marianna-u.ac.jp (N.F.); m-mine@marianna-u.ac.jp (M.M.); 3 Department of Pathology, St. Marianna University School of Medicine, Kawasaki 216-8511, Kanagawa, Japan; yoshiya.sugiura@marianna-u.ac.jp (Y.S.); j2koike@marianna-u.ac.jp (J.K.)

**Keywords:** non-small cell lung cancer, neoadjuvant chemoimmunotherapy, perioperative outcomes, thoracic surgery, pathologic response, neoadjuvant therapy, surgical feasibility

## Abstract

**Background:** Neoadjuvant chemoimmunotherapy has emerged as a standard option for resectable stage II–III non-small cell lung cancer (NSCLC); however, its impact on surgical feasibility, operative complexity, and perioperative outcomes remains incompletely characterized. We compared surgical feasibility, complexity, and perioperative outcomes across different neoadjuvant strategies. **Methods:** This single-center retrospective study included patients with stage II–III NSCLC who underwent curative-intent resection following neoadjuvant chemoimmunotherapy (neo-CIT), chemotherapy alone (neo-CT), or chemoradiotherapy (neo-CRT) during the same period. Surgical complexity was assessed using a four-level empirical grading scale. Perioperative outcomes, pathological response, and complications were compared among treatment groups using appropriate nonparametric statistical methods. **Results:** Twenty-four patients were included in the analysis (neo-CIT, n = 8; neo-CT, n = 12; neo-CRT, n = 4). R0 resection was achieved in 100%, 66.7%, and 75% of patients in the neo-CIT, neo-CT, and neo-CRT groups, respectively. Operative time, estimated blood loss, length of hospital stay, and overall postoperative complication rates did not differ significantly among groups. Thirty-day mortality was 0% in all groups, and 90-day mortality was 12.5% in the neo-CIT group and 0% in the neo-CT and neo-CRT groups. Surgical complexity scores were high across all cohorts and, when analyzed across the entire cohort, were not significantly associated with longer operative time (*p* = 0.16), greater blood loss (*p* = 0.83), or pathological response (*p* = 0.55). Neo-CIT was not associated with increased objective perioperative risk compared with neoadjuvant chemotherapy or chemoradiotherapy. **Conclusions:** In this single-institution exploratory analysis, neoadjuvant chemoimmunotherapy appeared to be surgically feasible and did not result in an apparent increase in perioperative risk compared with other neoadjuvant strategies. Although surgeons perceived increased operative complexity, objective perioperative risk remained acceptable when procedures were performed by experienced thoracic surgeons. However, given the limited sample size and lack of statistical power, these findings should be interpreted with caution and should not be considered evidence of equivalence.

## Simple Summary

Neoadjuvant chemoimmunotherapy is increasingly used before surgery for patients with resectable stage II–III non-small cell lung cancer. Although this treatment can improve cancer control, thoracic surgeons remain concerned that inflammation and tissue changes caused by immunotherapy may make subsequent surgery more difficult. This study aimed to determine whether surgery after chemoimmunotherapy is more technically challenging or associated with worse perioperative outcomes than surgery after chemotherapy or chemoradiotherapy. We retrospectively compared surgical complexity, complete resection, operative time, blood loss, hospital stay, and postoperative complications among these treatment groups. Although surgeons perceived surgery after chemoimmunotherapy as more technically challenging, this did not translate into worse objective surgical outcomes, and no deaths occurred within 30 days after surgery. These findings support the surgical feasibility of neoadjuvant chemoimmunotherapy, although confirmation in larger studies is needed.

## 1. Introduction

Lung cancer remains the leading cause of cancer-related morbidity and mortality worldwide [1,2]. Non-small cell lung cancer (NSCLC) accounts for approximately 80% of all lung cancer cases, and only about 30% of NSCLC patients present with resectable disease at initial diagnosis [3]. Surgical resection remains the cornerstone of curative-intent treatment for NSCLC, particularly for patients with stage II–III disease. The 5-year overall survival (OS) rate following curative resection is approximately 60–70% in early-stage disease, but declines to about 35% in stage II–III disease and to approximately 20% in more advanced stages [4]. These suboptimal outcomes are largely attributable to occult micrometastatic disease present at the time of surgery, which contributes to postoperative recurrence and distant metastasis [5].

To address this limitation, neoadjuvant systemic therapies have been increasingly incorporated into the management of resectable NSCLC. Building on the success of immune checkpoint inhibitors (ICIs) in advanced-stage disease, agents targeting programmed death protein 1 (PD-1) and programmed death ligand 1 (PD-L1) have been actively investigated in the neoadjuvant and perioperative settings. Neoadjuvant immunotherapy is biologically appealing, as treatment is delivered in the presence of an intact tumor microenvironment and high tumor antigen burden, which may enhance antitumor immune priming and improve control of micrometastatic disease [6,7,8]. However, robust and standardized approaches to evaluate therapeutic efficacy in this setting remain incompletely established.

Recent phase III trials evaluating neoadjuvant or perioperative regimens for resectable, locally advanced NSCLC have demonstrated meaningful clinical benefit. These regimens typically combine ICIs with chemotherapy, followed by adjuvant ICI monotherapy or standard postoperative management. Compared with chemotherapy alone, ICI-based combination strategies have been associated with higher rates of pathological complete response, improved resectability, and superior event-free and OS outcomes. Subgroup analyses suggest that these benefits may be influenced by PD-L1 expression and depth of pathologic response [9,10,11,12,13,14,15,16,17,18]. Based on these findings, neoadjuvant or perioperative chemoimmunotherapy has emerged as a standard treatment option for patients with resectable stage II–III NSCLC.

Despite these advances, important clinical questions remain unanswered. While pathological response and survival endpoints have been the primary focus of most trials, the balance between therapeutic efficacy and potential toxicity must be carefully considered. This is particularly important because preoperative systemic therapies may affect a patient’s eligibility for lung cancer resection and influence perioperative outcomes [19]. Furthermore, it is essential to continuously evaluate potential surgical challenges associated with novel treatments administered before planned surgical resection [20,21,22,23,24].

In this retrospective, single-institution study, we systematically assessed surgical feasibility and perioperative outcomes in patients with stage II–III NSCLC undergoing lung cancer resection. We focused on surgical approach, timing, completeness of resection, surgical complexity, and perioperative morbidity and mortality. Outcomes following neoadjuvant chemoimmunotherapy were compared with those observed after neoadjuvant chemotherapy or chemoradiotherapy, as well as with primary surgical resection performed during the same study period. Through this analysis, we aim to clarify the real-world surgical implications of contemporary neoadjuvant treatment strategies for resectable NSCLC.

## 2. Methods

### 2.1. Study Design and Patients

This was a single-center, retrospective observational study of patients with resectable NSCLC who underwent curative-intent surgery following neoadjuvant therapy at the St. Marianna University School of Medicine Hospital (Kawasaki, Japan). Between January 2019 and December 2024, surgical patients with stage II–III NSCLC received neoadjuvant treatment and were classified into three groups based on the neoadjuvant regimen: (1) neoadjuvant chemoimmunotherapy (neo-CIT) consisting of platinum-based chemotherapy combined with an ICI (nivolumab or pembrolizumab) for up to four cycles; (2) neoadjuvant chemotherapy (neo-CT) consisting of platinum-based doublet chemotherapy alone for up to four cycles; and (3) neoadjuvant chemoradiotherapy (neo-CRT) consisting of concurrent chemotherapy for up to four cycles and thoracic radiotherapy at a total dose of 40–60 Gy. Treatment strategy was determined through multidisciplinary discussion based on clinical stage, patient condition, and evolving institutional practice patterns.

This retrospective surgical cohort included patients with stage II–III NSCLC who received neoadjuvant therapy and subsequently underwent curative-intent pulmonary resection. Patients who initiated neoadjuvant therapy but did not proceed to surgery were not systematically captured in the surgical database and therefore could not be reliably identified. Accordingly, the present study does not evaluate treatment completion or treatment-to-surgery attrition.

The study was conducted in accordance with the Declaration of Helsinki and approved by the Institutional Review Board of St. Marianna University School of Medicine (protocol code 2233; date of approval: 11 March 2022).

### 2.2. Surgical Procedures and Complexity

Surgical resection was basically performed within 3–8 weeks after the completion of neoadjuvant therapy and conducted by a board-certified thoracic surgeon either as the primary surgeon or as a supervising assistant. The surgical approach was either video-assisted thoracoscopic surgery (VATS) or thoracotomy, according to the surgeon’s preference. Assessment of surgical complexity was the primary focus of this study. Intraoperative dissection difficulty was independently evaluated by three board-certified thoracic surgeons using the following four-grade scale proposed by Sepesi et al. [13]: (1) easier than normal tissue dissection, defined as tissues that separate easily, predominantly with blunt dissection; (2) normal tissue dissection, comparable to routine anatomical lobectomy, requiring both blunt and sharp dissection; (3) difficult dissection due to inflammation, characterized by partially obliterated tissue planes with predominantly sharp dissection required; and (4) very complex dissection, in which tissue planes are completely obliterated, resembling fibrosis or post-radiation changes.

In cases where a numerical score was not explicitly documented, operative records were reviewed, and the final dissection difficulty score was determined by consensus among the three thoracic surgeons.

### 2.3. Pathological Evaluation

All resected specimens were reviewed by three board-certified pathologists certified by the Japanese Society of Pathology. Pathologic staging was performed according to the 9th edition of the TNM classification system. Pathological response was assessed according to the IASLC Multidisciplinary Recommendations for Pathologic Assessment of Lung Cancer Resection Specimens After Neoadjuvant Therapy [25]. The percentage of residual viable tumor cells was evaluated within the tumor bed. Pathological response was categorized as pathologic complete response (pCR), defined as the absence of viable tumor cells in both the resected primary tumor and sampled regional lymph nodes; major pathologic response (MPR), defined as 10% or fewer residual viable tumor cells in the primary tumor; and non-major pathologic response (non-MPR), defined as more than 10% residual viable tumor cells. Pathological nodal response was evaluated separately based on the presence or absence of residual viable tumor cells in the resected lymph nodes.

R0 resection is defined as microscopically margin-negative resection, R1 as microscopic residual disease, and R2 as macroscopic residual disease.

### 2.4. Perioperative Surgical Outcomes

Evaluated outcomes included surgical complexity, feasibility, perioperative outcomes, and pathological response. The parameters comprised the R0 resection rate; operative time, estimated blood loss, and red blood cell transfusion; postoperative complications classified according to the Clavien–Dindo classification and length of stay; and pathological downstaging and response rates. Thirty- and 90-day mortality are reported separately.

### 2.5. Statistical Analysis

All statistical analyses were conducted using EZR (Saitama Medical Center, Jichi Medical University, Saitama, Japan), a graphical user interface for R. Owing to the small sample size and non-normal distribution of continuous variables, nonparametric tests were applied. Continuous variables, including age, tumor size, forced expiratory volume in 1 s (FEV1), maximum standardized uptake value (SUVmax), operation time, estimated blood loss, and length of hospital stay, were compared among the three groups using the Kruskal–Wallis test. Categorical variables, including sex, Eastern Cooperative Oncology Group (ECOG) performance status, smoking status, histologic subtype, clinical stage, nodal status, radiologic response, transfusion requirement, complications, and pathological findings, were compared using the Fisher–Freeman–Halton exact test, an extension of Fisher’s exact test for multi-group categorical data. Owing to small cell counts (n < 5), *t*-tests and chi-square tests were not performed. All *p*-values < 0.05 were considered statistically significant.

## 3. Results

### 3.1. Patients

**Figure 1:**
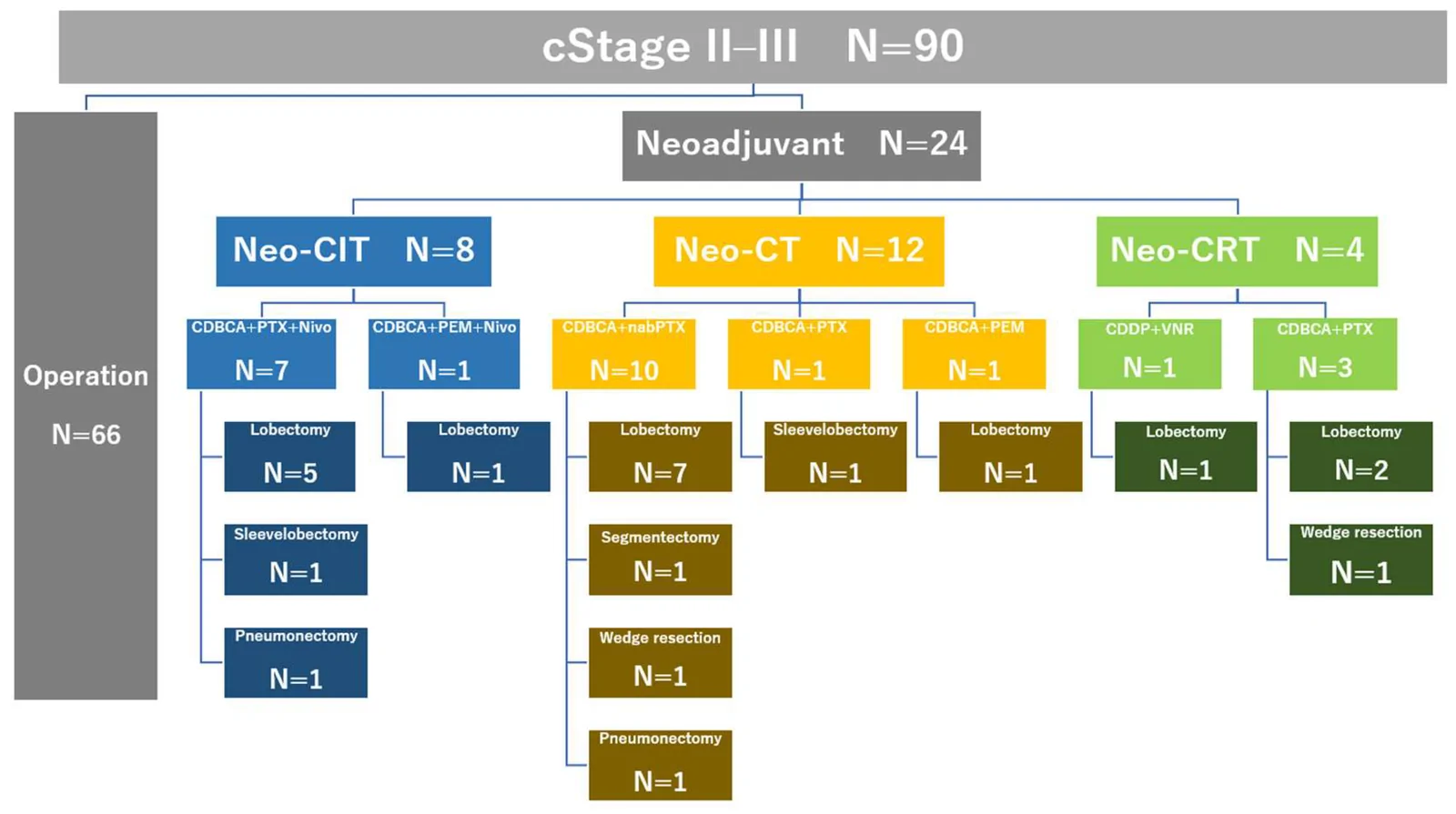
Patient flow and surgical management following neoadjuvant therapy. Of 90 patients with clinical stage II–III NSCLC evaluated during the study period, 66 underwent upfront surgical resection and 24 received neoadjuvant treatment followed by surgery. The neoadjuvant cohort included patients treated with chemoimmunotherapy (neo-CIT; n = 8), chemotherapy alone (neo-CT; n = 12), and chemoradiotherapy (neo-CRT; n = 4). The figure details the specific neoadjuvant regimens administered in each neoadjuvant group and the subsequent surgical procedures performed (lobectomy, sleeve lobectomy, segmentectomy, wedge resection, or pneumonectomy).

Between January 2019 and December 2025, a total of 90 patients with stage II–III NSCLC underwent surgical resection. Among these, 24 patients who had received neoadjuvant treatment were eligible for inclusion in the present study. Of these 24 patients, 8 patients received neo-CIT, 12 received neo-CT, and 4 received neo-CRT. Details of the neoadjuvant regimens and subsequent surgical procedures are presented in Figure 1. Baseline clinicopathologic characteristics of patients in the three treatment groups are summarized in Table 1.

### 3.2. Tumor Responses After Neoadjuvant Treatment

Radiologic response rates after at least one cycle of preoperative neoadjuvant therapy, assessed by CT performed within one month before surgery, are shown in Table 2. In the neoadjuvant chemoimmunotherapy group, among the 8 evaluable patients, one patient (12.5%) achieved a complete response (CR), five patients (62.5%) achieved a partial response (PR), and two patients (25.0%) had stable disease (SD). One case of progressive disease (PD) was observed in neo-CRT. In the neoadjuvant chemotherapy and neoadjuvant chemoradiotherapy groups, CR was observed in zero patients in both groups, while PR was observed in 10 (83.3%) and three (75%) patients, respectively. No statistically significant differences in radiologic response rates were identified between the treatment groups (*p* = 0.213).

### 3.3. Surgical Procedures and Approach

**Figure 2:**
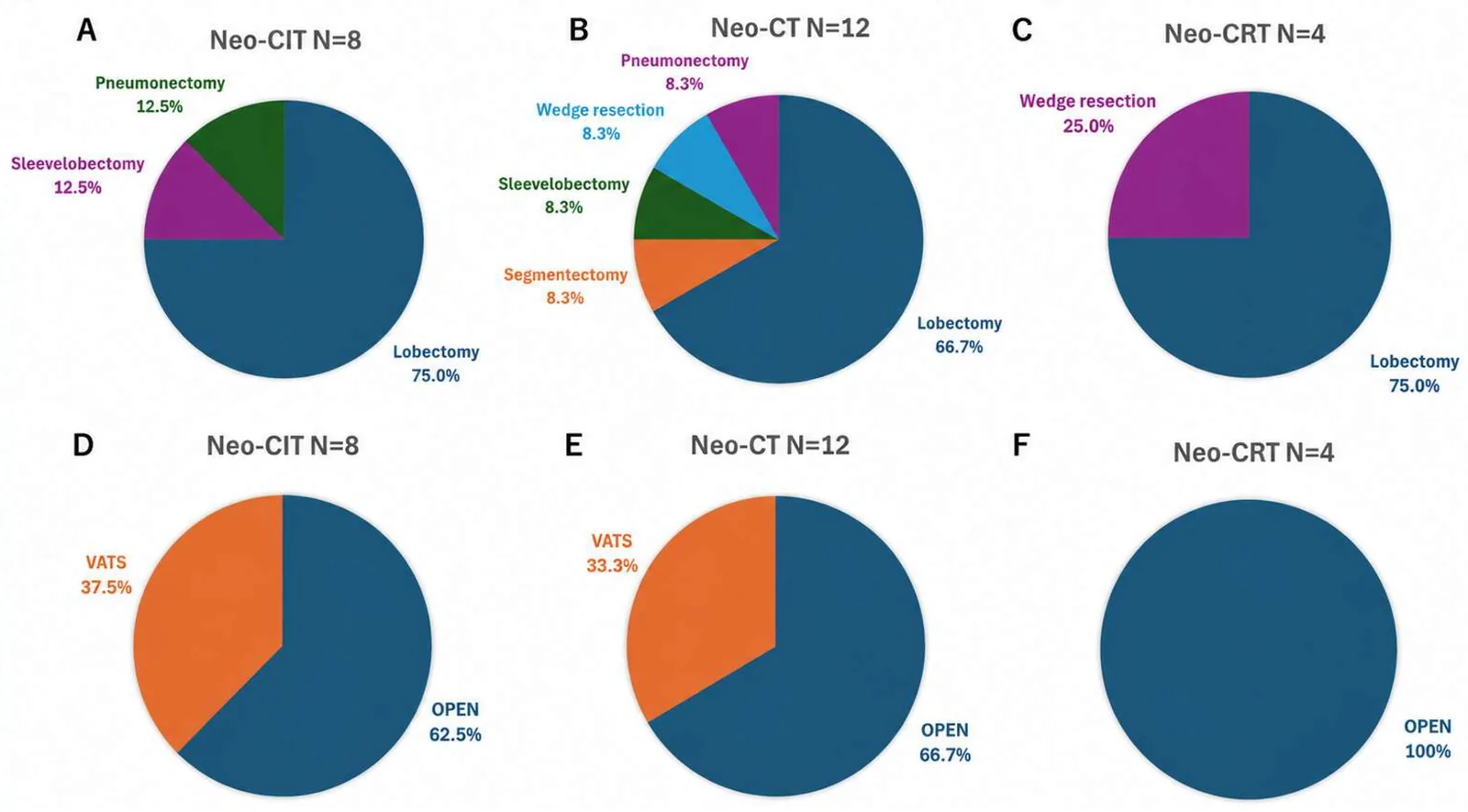
Surgical procedures and surgical approach according to the neoadjuvant treatment group. Pie charts illustrate the distribution of resection types (panels (**A**–**C**)) and operative approaches (panels (**D**–**F**)) among patients who received neoadjuvant therapy, including neoadjuvant chemoimmunotherapy (neo-CIT; n = 8), neoadjuvant chemotherapy (neo-CT; n = 12), and neoadjuvant chemoradiotherapy (neo-CRT; n = 4). Panels (**A**–**C**) display the proportions of lobectomy and other procedures (segmentectomy, sleeve lobectomy, wedge resection, and pneumonectomy) within each treatment group. Panels (**D**–**F**) depict the relative use of open thoracotomy versus video-assisted thoracoscopic surgery (VATS) in each group, with all patients in the neo-CRT group undergoing open thoracotomy.

Figure 2 summarizes the surgical procedures and approaches across the three groups. The proportions of patients undergoing lobectomy in the neo-CIT, neo-CT, and neo-CRT groups were 75%, 66.7%, and 85.7%, respectively. Pneumonectomy was performed in 12.5% of patients in the neo-CIT group and 8.3% of those in the neo-CT group, whereas no pneumonectomies were performed in the neo-CRT group. Sublobar resection including segmentectomy and wedge resection were performed in two of 12 patients (16.6%) in the neo-CT group and in one patient (25.0%) in the neo-CRT group. Sleeve lobectomy was performed in one of eight patients (12.5%) in the neo-CIT group and in one of 12 patients (8.3%) in the neo-CT group.

Regarding surgical approach, thoracotomy was performed in five patients (62.5%) and VATS in three patients (37.5%) in the neo-CIT group. In the neo-CT group, thoracotomy was performed in eight patients (66.7%) and VATS in four patients (33.3%). All patients in the neo-CRT group (100%) underwent thoracotomy.

### 3.4. Perioperative Outcomes

Perioperative outcomes for the three groups are summarized in Table 3. R0 resection was achieved in all patients in the neo-CIT group (8 of 8, 100%), in eight of 12 patients (66.7%) in the neo-CT group, and in three of 4 patients in the neo-CRT group (3 of 4, 75%). Two patients had microscopically positive parenchymal margins due to spread through airspaces.

The median interval from the final dose of neoadjuvant therapy to surgery was 44.5 days (range: 20–115) in the neo-CIT group, 33 days (range: 18–154) in the neo-CT group, and 23.5 days (range: 14–56) in the neo-CRT group. The prolonged intervals of 115 and 154 days were due to delays in surgery to allow recovery from severe adverse events associated with neoadjuvant therapy.

The median operative time was 298 min (range: 127–349) in the neo-CIT group, 276 min (range: 154–432) in the neo-CT group, and 284 min (range: 118–443) in the neo-CRT group, with no significant differences among the groups. The median estimated blood loss was 428.0 mL (range: 30–1760) in the neo-CIT group, 211.8 mL (range: 0–630) in the neo-CT group, and 214.0 mL (range: 34–515) in the neo-CRT group, with no significant differences among groups.

The median length of stay was 14 days (range: 5–40) in the neo-CIT group, 12 days (range: 6–113) in the neo-CT group, and 15 days (range: 3–20) in the neo-CRT group.

**Figure 3:**
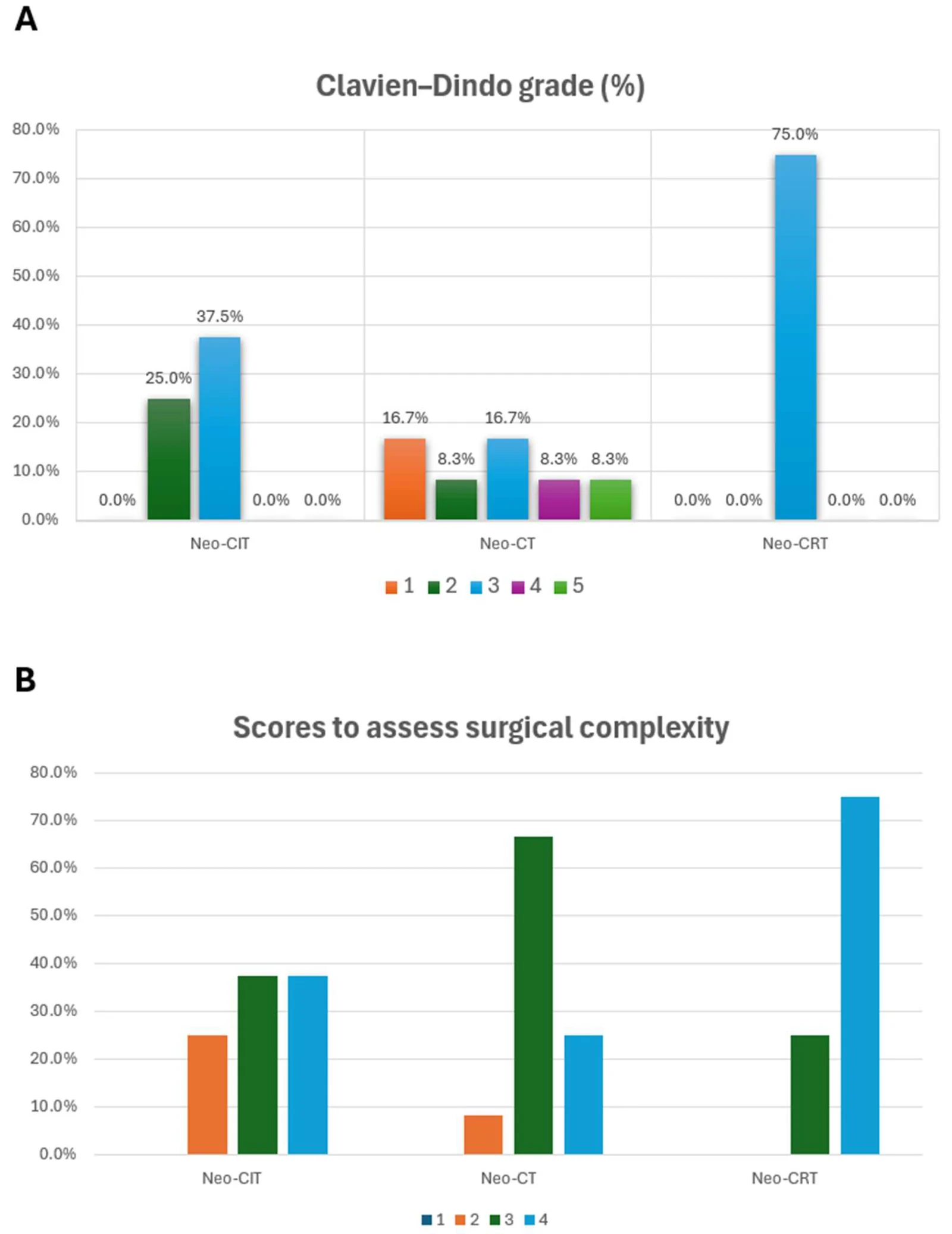
Postoperative morbidity and surgeon-assessed operative complexity according to neoadjuvant treatment group. (**A**) Distribution of postoperative complications graded according to the Clavien–Dindo classification (grades 1–5), presented as percentages within each neoadjuvant group (neo-CIT, neo-CT, and neo-CRT). (**B**) Distribution of surgeon-assessed surgical complexity scores (1–4), shown as percentages within each neoadjuvant group (neo-CIT, neo-CT, and neo-CRT).

Overall postoperative complication rates for neo-CIT, neo-CT, and neo-CRT groups were 62.5% (5/8), 58.3 (7/12), and 75% (3/4), respectively. Severe complications of Clavien–Dindo grade ≥ 3 occurred in three patients (37.5%) in the CIT group, four patients (33.3%) in the CT group, and three patients (75%) in the neo-CRT group (Figure 3A). The 30-day mortality rate was 0% in all three groups. The 90-day mortality rate was 12.5% (1/8) in the neo-CIT group, and 0% in both the neo-CT (0 of 12) and the neo-CRT (0 of 4) groups. The patient in the neo-CIT group had an uneventful postoperative course and was discharged from the hospital but subsequently died at home from interstitial pneumonitis considered to be an immune-related adverse event. Pulmonary complications occurred in nine patients (37.5%), including prolonged air leak (lasting >5 days) in six patients (25%) and perioperative pneumonitis in two patients (8.3%). In addition, one patient developed pulmonary edema requiring mechanical ventilation, which subsequently resolved in the neo-CIT group. Atrial fibrillation occurred in one patient in the neo-CRT group, and RBC transfusion was required in four patients among groups. One patient in the neo-CT group developed a bronchial stump fistula that progressed to severe empyema and resulted in death on postoperative day 170. Importantly, the postoperative death occurring on day 170 is now clearly distinguished from 30- and 90-day mortality and is not classified as perioperative mortality within these predefined time windows.

### 3.5. Pathologic Response

As summarized in Table 2, pathological CR was achieved in one patient (12.5%) in the neo-CIT group, two patients (16.7%) in the neo-CT group, and one patient (25.0%) in the neo-CRT group, with no statistically significant differences observed among the groups. In contrast, pathological nodal downstaging to N1 or N0 was observed in all patients in the neo-CRT group, representing a statistically significant finding.

### 3.6. Surgical Complexity

The distribution of the surgical complexity scores according to treatment group is shown in Figure 3B. The median complexity score was 3 (range, 2–4) in the neo-CIT group, 3 (range, 2–4) in the neo-CT group, and 4 (range, 2–4) in the neo-CRT group, with no statistically significant differences among groups (*p* = 0.249). Overall, surgeons rated 21 of 24 operations (87.5%) as having a complexity score of 3 or 4, indicating procedures that were more complex than a typical lobectomy for stage I disease. Notably, 17 of 24 operations (70.8%) had an operative duration exceeding 4 h, including five cases in the neo-CIT group, nine in the neo-CT group, and three in the neo-CRT group.

Among resections with complexity scores of 2, 3, and 4, the median operative times were 238, 300, and 286 min, respectively, and the median estimated blood losses were 140, 241, and 268 mL, respectively. Surgical complexity was not associated with longer operative time (*p* = 0.16), greater blood loss (*p* = 0.83), or pathological response (*p* = 0.55). These findings suggested that the complexity score primarily reflects individual surgeons’ subjective assessments of intraoperative difficulty.

## 4. Discussion

Our perioperative analysis demonstrated that 30- and 90-day mortality, Clavien–Dindo-graded morbidity, surgical outcomes, tumor response, and surgical complexity were broadly comparable among the neo-CIT, neo-CT, and neo-CRT groups in this single-institution retrospective cohort treated during the same era. These findings suggest that neo-CIT does not adversely affect objectively assessed postoperative outcomes when compared with neo-CT or neo-CRT in patients undergoing thoracic surgery. Nevertheless, several aspects of neoadjuvant immunotherapy and perioperative management warrant further consideration.

The introduction of neoadjuvant immunotherapy has substantially reshaped the treatment paradigm for resectable locally advanced NSCLC, with surgical resection rates remaining a central concern. In contemporary neoadjuvant trials, resectability is determined by the surgeon at enrollment; therefore, patients are enrolled with the expectation that surgical resection with curative intent, including achievement of R0 resection, will be feasible. Across published trials, reported resection rates range from approximately 83 to 95%, with R0 resection rates of 89 to 95% [9,10,11,12]. Although certain determinants of resection rates, such as patient selection, may be optimized, social circumstances, changes in patient preference, or unforeseen clinical events are inherently unpredictable. Consequently, a dropout rate of less than 10% is generally considered acceptable in neoadjuvant trials and is consistent with the findings of the present study.

In addition, the impact of neoadjuvant therapy on postoperative outcomes, particularly in the context of minimally invasive approaches including VATS and robotic-associated surgery, remains incompletely defined. Prior studies have reported that neoadjuvant therapy is associated with longer operative times but does not significantly increase conversion rates to thoracotomy or major postoperative complications [26,27]. These findings are supported by large prospective trials, including CheckMate 816, in which neoadjuvant chemoimmunotherapy with nivolumab was associated with increased operative time, reflecting greater surgical complexity, without a corresponding increase in conversion to open surgery [9]. Such observations likely reflect both treatment-related tissue changes, including tumor fibrosis and hilar adhesions, and the increasing expertise of thoracic surgeons in managing technically demanding minimally invasive procedures following systemic therapy. Consistent with these reports, our study demonstrated no significant differences in overall postoperative complications or length of hospital stay among the three treatment groups [23,24,26,27,28].

Concerns regarding operative complexity following immunotherapy emerged early in surgical experience, largely driven by anecdotal reports describing altered tissue quality and obscured anatomical planes. In the present study, operative time and perioperative outcomes were comparable between neoadjuvant chemotherapy alone and neoadjuvant chemoimmunotherapy, consistent with real-world evidence demonstrating similar safety profiles for both approaches [29]. Although neo-CIT has been associated with improved pathological responses, some reports have noted a modest increase in selected high-grade adverse events, including atrial fibrillation, highlighting the importance of careful patient selection [30,31].

To better capture surgeons’ intraoperative impressions, we applied an empirical surgical complexity grading system. The surgical complexity score used in this study remains subjective and not formally validated, and the lack of correlation with operative time, blood loss, or pathological response suggests that it primarily reflects surgeons’ intraoperative perception rather than objective surgical burden. Importantly, perceived operative complexity likely reflects not only treatment-related changes such as fibrosis and adhesions but also baseline disease characteristics, including tumor size, nodal involvement, and desmoplastic reaction. Collectively, these findings suggest that surgery following neoadjuvant immunotherapy may be feasible without a clear increase in objective perioperative risk when appropriate surgical judgment and technical expertise are applied. These findings provide descriptive insight into the technical challenges encountered during surgery following neoadjuvant therapy; however, given the small sample size and the exploratory nature of the analysis, no definitive conclusions regarding comparative perioperative risk can be drawn.

This study has several limitations. This study should be interpreted primarily as a descriptive and exploratory assessment of surgical feasibility and perioperative experience rather than a comparative effectiveness study. First, its retrospective, single-center design is inherently subject to selection bias and unmeasured confounding, which cannot be fully eliminated. Importantly, this study included only patients who ultimately underwent surgical resection after neoadjuvant therapy. Patients who initiated neoadjuvant treatment but did not proceed to surgery because of disease progression, treatment-related toxicity, functional decline, patient preference, unresectability, or other reasons were not systematically captured in the surgical database. Therefore, treatment completion, treatment-to-surgery attrition, and the overall probability of proceeding to surgery after neoadjuvant therapy could not be assessed. Accordingly, the present findings apply only to the selected population of patients who ultimately underwent surgery and should not be extrapolated to all patients initiating neoadjuvant therapy.

In addition, the surgical complexity score was also retrospectively assigned, surgeons were not blinded to treatment group, and in some cases the score was reconstructed from operative reports. Therefore, this measure should be regarded as an exploratory and hypothesis-generating measure of surgeon-perceived operative difficulty rather than a validated objective assessment of operative difficulty. Perceived operative complexity likely reflects not only treatment-related changes but also baseline disease characteristics, including tumor size, nodal involvement, and desmoplastic reaction.

Finally, the relatively small sample size, particularly within the neoadjuvant cohorts and individual treatment subgroups, limits statistical power and generalizability and precluded adjusted or multivariable analyses. Accordingly, residual confounding and selection bias cannot be excluded, and therefore differences (or lack of differences) among groups should not be interpreted as treatment effects. Likewise, the absence of statistically significant differences should not be interpreted as evidence of equivalence between treatment strategies. Larger prospective studies are warranted to validate these findings and to clarify long-term oncologic outcomes following neoadjuvant therapy.

In conclusion, although neoadjuvant immunotherapy may alter intraoperative tissue characteristics and increase surgeon-perceived technical complexity, the present findings should be interpreted with caution. Among selected patients with stage II–III NSCLC who ultimately underwent surgical resection after neoadjuvant therapy, surgery was completed across all three treatment groups despite frequent surgeon-perceived operative complexity. This small exploratory series did not provide definitive evidence of differences in reported perioperative outcomes among the treatment groups. However, the limited sample size, baseline heterogeneity, nonrandom treatment selection, and lack of adjustment preclude conclusions regarding comparative safety or equivalence. Accordingly, these findings should be regarded as unadjusted, exploratory, and hypothesis-generating and require validation in larger prospective cohorts.

## 5. Conclusions

Neoadjuvant chemoimmunotherapy appeared surgically feasible without an apparent increase in perioperative risk compared with other neoadjuvant strategies, despite greater surgeon-perceived operative complexity. However, given the small sample size, baseline heterogeneity, lack of adjustment and limited statistical power, these findings should be interpreted with caution and should not be considered evidence of equivalence.

## Figures and Tables

**Table 1:** Baseline clinicopathological characteristics of the study population.

Variable	Category	Neo-CIT (n = 8)	Neo-CT (n = 12)	Neo-CRT (n = 4)	*p*-Value
Age (years), mean ± SD		66.4 ± 4.6	68.8 ± 9.9	69.0 ± 7.7	0.334
Sex, n (%)	Male	7 (87.5%)	8 (66.7%)	2 (50.0%)	0.603
	Female	1 (12.5%)	4 (33.3%)	2 (50.0%)	
ECOG PS, n (%)	0	4 (50.0%)	5 (41.7%)	2 (50.0%)	0.014
	1	4 (50.0%)	7 (58.3%)	0 (0.0%)	
	2	0 (0.0%)	0 (0.0%)	2 (50.0%)	
Smoking status, n (%)	Never smoker	3 (37.5%)	3 (25.0%)	0 (0.0%)	0.228
	Former/current smoker	5 (62.5%)	9 (75.0%)	4(100.0%)	
FEV1 (L), mean (range)		2.4 (2–3)	1.9 (1–3)	1.6 (1–2)	0.076
Histology, n (%)	Squamous	4 (50.0%)	6 (50.0%)	1 (25.0%)	1.000
	Nonsquamous	4 (50.0%)	6 (50.0%)	3 (75.0%)	
Tumor size, mm		52.8 (27–96)	59.5 (22–100)	55.5 (35–73)	0.440
Nodal status	N0	3 (37.5%)	5 (41.7%)	0 (0.0%)	0.842
	N1	2 (25.0%)	2 (16.7%)	0 (0.0%)	
	N2a	1 (12.5%)	1 (8.3%)	0 (0.0%)	
	N2b	2 (25.0%)	4 (33.3%)	4 (100.0%)	
Clinical stage, n (%)	IIAB	3 (37.5%)	3 (25.0%)	0 (0.0%)	1.000
	IIIAB	5 (62.5%)	9 (75.0%)	4 (100.0%)	
SUVmax, mean ± SD		13.9 ± 3.6	14.5 ± 5.1	19.7 ± 9.7	0.815

neo-CIT, neoadjuvant chemoimmunotherapy; neo-CT, neoadjuvant chemotherapy; neo-CRT, neoadjuvant chemoradiotherapy; FEV1, forced expiratory volume during the first second; ECOG PS, Eastern Cooperative Oncology Group Performance Status; SUVmax, maximum standardized uptake value.

**Table 2:** Tumor responses following neoadjuvant treatment *.

Variable		Neo-CIT (n = 8)	Neo-CT (n = 12)	Neo-CRT (n = 4)	*p*-Value
**Radiologic response**					
Complete response		1 (12.5%)	0 (0.0%)	0 (0.0%)	0.213
Partial response		5 (62.5%)	10 (83.3%)	3 (75.0%)	
Stable disease		2 (25.0%)	2 (16.7%)	0 (0.0%)	
Progressive disease		0 (0.0%)	0 (0.0%)	1 (25.0%)	
**Pathological response**					
Pathological complete response		1 (12.5%)	2 (16.7%)	1 (25.0%)	0.495
Pathological T down staging		5 (62.5%)	5 (41.7%)	2 (66.7%)	0.650
Pathological N down staging (to N1 or N0)		4 (50.0%)	3 (25.0%)	4 (100%)	
Pathological staging	I	3 (37.5%)	4 (33.3%)	1 (25.0%)	1.000
	II	4 (50.0%)	2 (16.7%)	2 (50.0%)	0.161
	III	1 (12.5%)	6 (50.0%)	1 (25.0%)	0.158

* Based on Response Evaluation Criteria in Solid Tumors (RECIST) version 1.1 and classification. neo-CIT, neoadjuvant chemoimmunotherapy; neo-CT, neoadjuvant chemotherapy; neo-CRT, neoadjuvant chemoradiotherapy.

**Table 3:** Perioperative outcomes by neoadjuvant treatment group.

Variable	Neo-CIT (n = 8)	Neo-CT (n = 12)	Neo-CRT (n = 4)	*p*-Value
R0 resection	8 (100.0%)	8 (66.7%)	3 (75.0%)	0.117
R1 resection	0 (0.0%)	4 (33.3%)	1 (25.0%)	0.117
R2 resection	0 (0.0%)	0 (0.0%)	0 (0.0%)	1.000
Time from last neoadjuvant therapy to operation, median (range)	44.5 (20–115)	33.0 (18–154)	23.5 (14–56)	
30-Day mortality	0 (0%)	0 (0%)	0 (0%)	
90-Day mortality	1 (12.5%)	0 (0%)	0 (0%)	
Operation time (min), median (range)	298 (127–349)	276 (154–432)	284 (118–443)	0.970
Estimated blood loss (mL), median (range)	428.0 (30–1760)	211.8 (0–630)	214.0 (34–515)	0.230
Red Blood Cell transfusion	2 (25.0%)	1 (8.3%)	1 (25.0%)	0.537
Length of stay, days, median (range)	14 (5–40)	12 (6–113)	15 (3–20)	0.72
Complication (any)	5 (62.5%)	7 (58.3%)	3 (75.0%)	1.000
Clavien–Dindo grade ≥ 3	3 (37.5%)	4 (33.3%)	3 (75.0%)	1.000

neo-CIT, neoadjuvant chemoimmunotherapy; neo-CT, neoadjuvant chemotherapy; neo-CRT, neoadjuvant chemoradiotherapy.

## Data Availability

The data presented in this study are available from the corresponding author upon reasonable request. The data are not publicly available due to privacy and ethical restrictions. The datasets used and/or analysed during the current study are available from the corresponding author on reasonable request.

## Abbreviations

CR	complete response
ECOG PS	Eastern Cooperative Oncology Group performance status
FEV1	forced expiratory volume in 1 s
ICI	immune checkpoint inhibitor
MPR	major pathologic response
neo-CIT	neoadjuvant chemoimmunotherapy
neo-CT	neoadjuvant chemotherapy
neo-CRT	neoadjuvant chemoradiotherapy
NSCLC	non-small cell lung cancer
OS	overall survival
pCR	pathologic complete response
PD	progressive disease
PD-1	programmed death protein 1
PD-L1	programmed death ligand 1
PR	partial response
RBC	red blood cell
SD	stable disease
SUVmax	maximum standardized uptake value
TNM	Tumor–Node–Metastasis
VATS	video-assisted thoracoscopic surgery
